# A Review on Isolation, Characterization, Modification, and Applications of Proso Millet Starch

**DOI:** 10.3390/foods12122413

**Published:** 2023-06-19

**Authors:** Simmi Ranjan Kumar, Nuttinee Tangsrianugul, Manop Suphantharika

**Affiliations:** Department of Biotechnology, Faculty of Science, Mahidol University, Rama 6 Road, Bangkok 10400, Thailand; simmiranjankumar@gmail.com (S.R.K.); nuttineeta@gmail.com (N.T.)

**Keywords:** proso millet starch, extraction, characterization, modification, in vitro digestibility, application

## Abstract

Proso millet starch (PMS) as an unconventional and underutilized millet starch is becoming increasingly popular worldwide due to its health-promoting properties. This review summarizes research progress in the isolation, characterization, modification, and applications of PMS. PMS can be isolated from proso millet grains by acidic, alkaline, or enzymatic extraction. PMS exhibits typical A-type polymorphic diffraction patterns and shows polygonal and spherical granular structures with a granule size of 0.3–17 µm. PMS is modified by chemical, physical, and biological methods. The native and modified PMS are analyzed for swelling power, solubility, pasting properties, thermal properties, retrogradation, freeze–thaw stability, and in vitro digestibility. The improved physicochemical, structural, and functional properties and digestibility of modified PMS are discussed in terms of their suitability for specific applications. The potential applications of native and modified PMS in food and nonfood products are presented. Future prospects for research and commercial use of PMS in the food industry are also highlighted.

## 1. Introduction

Global production of cereal grains has reached record levels. Cereal grains play an important role in the human diet as a primary source of energy. The Food and Agriculture Organization (FAO) [1] reported in 2020 that the global production of cereal grains in 2019 reached a record high of 2715 million tons. At the same time, the global community is facing climatic changes, pollution, water scarcity, rising food costs, population growth, and other socioeconomic issues. These negative aspects can affect regional agricultural progress and limit grain production, leading to high food prices and serious food security concerns worldwide [2]. Moreover, smallholder farmers facing these conditions become economically vulnerable due to their limited resources and have difficulty maintaining their yields and profitability [3]. As a consequence of unfavorable global phenomena and their adverse impacts that constrain agricultural production, there is an urgent need among experts in nutrition and technology to identify a suitable cereal crop that could serve as a viable food source to address these challenges [4]. Under these circumstances, millets may be a nutritious option to supplement the nutritional needs of a growing world population in an uncertain global environment [5].

Millet belongs to the Poaceae family and is cultivated in subtropical and tropical regions of marginal drylands. Over 10,000 years ago, prior to the widespread consumption of wheat, maize, barley, and rice, this food item served as a staple for the people of that era. Currently, the most commonly cultivated species include proso millet (*Panicum miliaceum*), pearl millet (*Pennisetum glaucum*), and finger millet (*Setaria italica*) [6]. Millet is abundant in proteins, fats, carbohydrates, fiber, minerals, vitamins, and phenolic compounds [7]. Nutritionally, millet contains proteins (6–19%), carbohydrates (60–70%), fats (1.5–5%), minerals (2–4%), dietary fiber (12–20%), and various phytochemicals [8]. In addition, millet is gluten-free. This is desirable for people with celiac disease, and because of millet’s blood sugar-lowering properties, it is also effective in treating type II diabetes [8].

Proso millet (*Panicum miliaceum* L.) is also known as common millet, hog millet, Russian millet, and broomcorn millet in certain areas [9]. Proso millet is characterized by its adaptability to unfavorable environmental conditions (such as salt, drought, temperature, and pH). It also has a short lifecycle (about 12 weeks) and is grown in slightly acidic, saline, sandy, and low-fertility soils with limited nitrogen and carbon dioxide [10,11,12]. Proso millet contains carbohydrates (70–74%), proteins (9.4–9.9%), ash (1.2–3.8%), and fats (1.2–3.8%), along with a variety of phytochemicals and vital minerals [13].

Starch is a major constituent of millet and is divided into two types, namely, amylose and amylopectin. Based on the amylose content, millets are classified into nonwaxy (high amylose content) and waxy (low amylose content) [14]. Yang et al. [15] measured the range of starch content in nonwaxy (high amylose content) proso millet as 59–77% and for waxy millet as 55–69%. Starch serves as a crucial energy source for humans and is extensively utilized in the food and food-related industries. It is a renewable, biodegradable, economical, and natural material used to modify the textural properties of various foods. It can be modified into thickeners, stabilizers, and sweeteners, and can also serve as a water-retention agent [9].

A number of researchers conducted an analysis comparing various types of millet starches, but, unfortunately, they did not provide a thorough study of proso millet starch (PMS) [6,16,17]. According to Banger et al. [18], a comprehensive account of PMS, including its physiochemical and functional properties, modification, and applications, was presented. However, it was noted that more detailed information on isolation, digestibility, and recent advances in its applications is lacking.

Our goal is to fill this information gap by conducting a comprehensive study that includes the latest and detailed information on the isolation, physicochemical, functional, and morphological properties, modifiability, and in vitro digestibility of PMS and to investigate its potential applications in novel foods. Our research will provide detailed insights and contribute to the existing body of knowledge on this topic.

## 2. Isolation, Yield, and Composition of Proso Millet Starch

**Figure 1:**
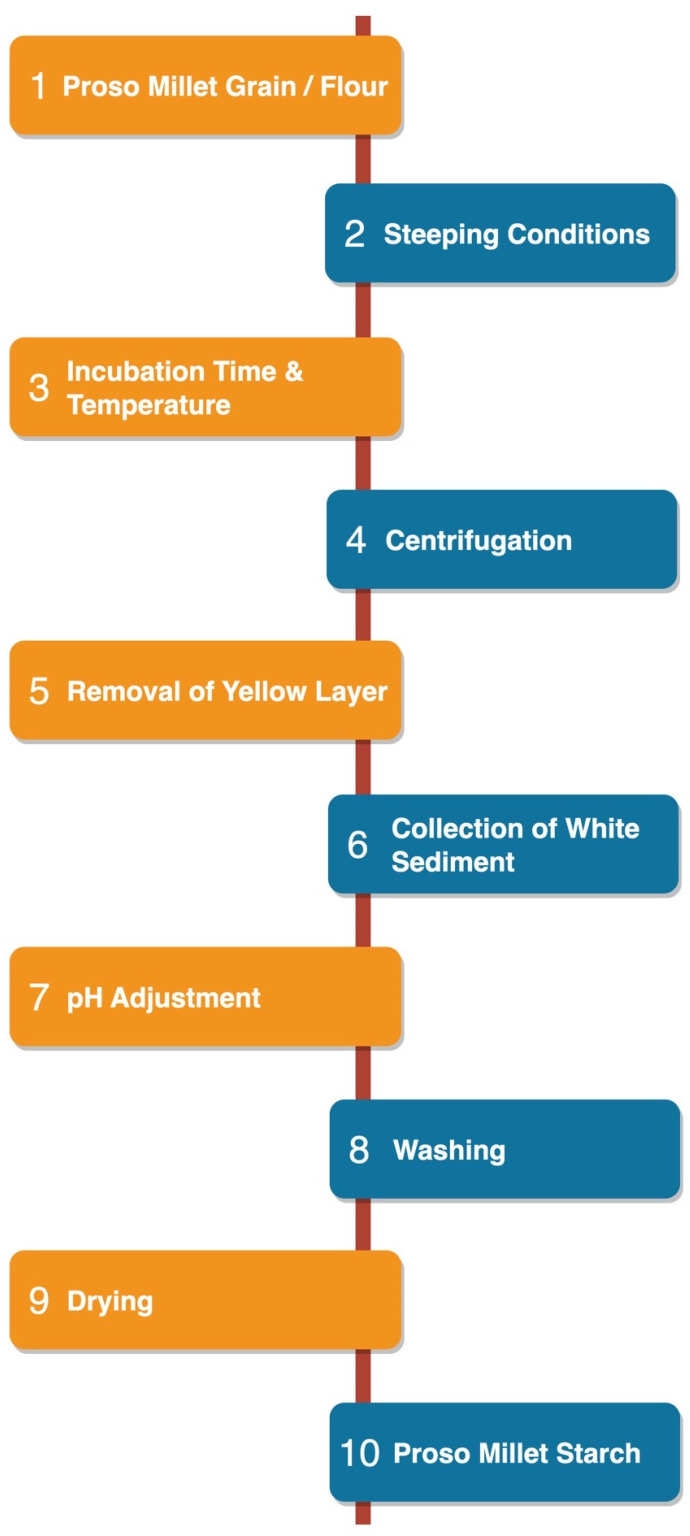
Isolation of proso millet starch.

The starch granules within proso millet grains exhibit strong binding affinity to the surrounding protein matrix. Various methods and chemical reagents are used to extract starch and solubilize the proteins in the grain [19]. Generally, starch extraction methods consist of three phases, i.e., fragmentation, cell disruption, and purification or separation [20]. Millet starch is usually isolated by the wet milling method. The grain or flour is soaked in an aqueous solution (water, alkali, or acid) for a certain time, depending on its chemical properties and composition [16]. The particular method of starch extraction (e.g., acidic, alkaline, or enzymatic) has a significant effect on starch yield. Starch isolation methods vary widely and depend on the inherent starch content of the grain and the initial soaking conditions (neutral, alkaline, or acidic) [21]. The procedure for isolating proso millet starch (PMS) is depicted in Figure 1.

In the alkaline steeping method, the grains are soaked with 0.3% sodium hydroxide (NaOH) solution for 24 h at 4 °C. The soaked grains are then ground to a wet slurry using a mill and then sieved through a 100-mesh sieve. After this, the samples are centrifugated at 3000 rpm for 15 min, the supernatant is removed, and the remaining contents are resuspended in water. This washing step is repeated for a total of 3 cycles; the slurry is then neutralized with hydrochloric acid (HCl). After washing/neutralization, the starch cake is dehydrated at 40 °C for 48 h [22]. In the acid steeping method of PMS extraction, the grains are soaked with 0.15% sulfur dioxide (SO_2_) solution for 48 h at 52 °C. The soaked grains are then crushed with a blender, sieved through a 40-mesh sieve, and washed with water. The residual material is then crushed using a mortar and pestle and filtered through a 200-mesh sieve and then through a 270-mesh sieve. The residue is again washed with water, filtered through a Buchner funnel with No. 2 Whatman filter paper, centrifuged, and dried overnight at 45 °C [6].

To extract starch from proso, pearl, kodo, foxtail, little, and barnyard millets, a practically neutral solution (pH 6.5) containing a minute amount of either sodium azide (0.01%) or mercury (II) chloride (0.01%) is used to prevent bacterial growth and inhibit amylase activity [6]. However, it should be noted that the use of sodium azide and/or mercury (II) chloride can cause serious health problems if ingested. A small amount of sulfur dioxide (0.5 g/L) and lactic acid (0.15 g/L) are added to isolate starch from proso millet in the acid steeping procedure. Similarly, the addition of a small amount of NaOH (0.1%) and sodium borate buffer comprising SDS (0.5%) and Na_2_S_2_O_5_ (0.5%) are used to isolate starch from these treatments. The use of these isolating solutions can significantly impact the chemical composition and characteristics of the extracted starch. In a comparative study between acid and alkaline steeping, acid steeping has a higher residual protein content (4.3%) than alkaline steeping (0.7%) [23].

The amount of millet starch obtained and the resulting chemical compositions differ significantly in the studies presented in Table 1. Millet contains about the same amount of starch as other cereal grains. The millet starch usually contains 20–30% amylose and 70–80% amylopectin. The presence of impurities in millet starch grains has significant implications for achieving some desired functional objectives [16]. For example, millet starch contains mainly nonpolar and polar lipids. The majority (89%) of the overall lipid content is attributed to polar phospholipids, whereas the rest primarily comprises nonpolar triglycerides [24]. These lipids can combine with the amylose component of starch to form complexes, which can lead to a decrease in the starch’s swelling capacity and flowability. This is caused by the lipids’ hydrophobic bonds and cohesive nature [16].

## 3. Morphology and Crystallinity of Proso Millet Starch

The size of starch granules in millet varies depending on the plant species. Despite being generally spherical and polygonal in shape (as indicated in Table 2), the dimensions of these granules range from 0.3–17 µm. The polygonal shapes are also larger and have more indentations than the spherical shapes [25], and the morphology of the starch is strongly influenced by its treatment and/or biomodification [30]. In addition, differences in particle size of PMS obtained from proso millet grown in different regions can be due to local environmental aspects. An increase in altitude and reduced mean temperature can lead to bigger granules [26]. Additionally, the morphology of starch is influenced by the arrangement of starch granules inside the endosperm of the grain [31]. Cavities are dispersed randomly throughout the entire outer layer of the starch granules due to surface pores and protein bodies. These pores are connected to the central cavity of the granules, enabling specific molecules from the external environment to penetrate the granules [16]. From a starch modification perspective, this phenomenon is helpful. These pores allow OH ions or water to enter the granules, destroying the amylose-containing amorphous region. Consequently, the restrictive qualities of amylose are reduced, leading to enhanced starch swelling and hydration properties [32].

Millet starches are semi-crystalline and are similar to other starches that contain both crystalline and amorphous regions. Millet exhibits typical A-type polymorphic diffraction patterns [16]. The relative crystallinity observed for native starch is 35.7% with a diffraction peak at 2θ values of 15.3–23.1° for a single peak and about 17°–8° for a double peak [36]. Sun et al. [22] observed that the native starch of the proso millet exhibited A-type X-ray diffraction patterns with 2θ of 15°, 17°, 18°, and 23.5°, confirming a previous report by Kim et al. [34], which also confirmed an A-type diffraction pattern for PMS. In a 2019 study, the relative crystallinity of PMS was measured to range from 37.6% to 38.4% [14]. The differences in the degree of relative crystallinity can be attributed to a variety of factors, including the biological origin, plant variety, composition of amylose and amylopectin, conditions during cultivation, and maturity stage of the parent plant at the time of harvest [37]. Impurities present in the starch, such as other millet constituents, result in a shift of the peaks and a decline in intensity. This is because the occurrence of impurities alone increases the size of the amorphous region compared to the crystalline portion [38]. These general differences in starch granules all affect the degree of crystallinity, and due to the absence of amylose, this occurs without affecting the granular size [39]. A stable crystalline structure for starch is formed by long amylopectin chains, in contrast to the less stable shorter amylopectin chains, which are easily broken down by high temperatures [24]. Food processing techniques, such as milling, frequently cause damage to the physical structure of starch. The crystalline amylopectin is transformed into amorphous amylopectin during these processes, and the resulting material develops low-molecular-weight fractions. These changes in crystallinity affect food functionality [16].

## 4. Physiological and Functional Properties

### 4.1. Swelling Power and Solubility

With an appropriate quantity of water, the starch is subjected to heating, causing the granules to absorb moisture and undergo swelling. In this process, the components of the starch granule are leached out and largely dissolved in the form of amylose. Eventually, the swollen starch granules break down and disintegrate when they continue to be exposed to high temperatures. This activity is influenced by several factors, including the physical associations of the chemical components in the granules, the molecular structure of amylose and amylopectin, the intrinsic phosphorus groups, and the restricting entanglement of the lipid–amylose complex [40]. Starch granules undergo swelling when exposed to temperatures between 50–90 °C in the presence of water. Studies have shown that the swelling power (SP) of millet is lower compared to that of rye, potato, and wheat. This indicates that millet starch has greater resistance towards swelling due to its relatively strong binding force between the granules **[16]**. Much research has been conducted on SP of PMS, and some representative results are presented below. Singh and Adedeji [28] studied the SP of PMS at different temperatures (70–90 °C) and recorded the percentage range of their size changes, i.e., native starch (4.69–24.99%), acid-modified starch (4.94–21.26%), and hydrothermally modified starch (5.29–10.37%). At 95 °C, Xiao et al. [41] studied the SP of native PMS (13.77 g/g) and PMS with proanthocyanidins (14.15–19.83 g/g). Wu et al. [29] reported that the SP of PMS in their research was greater than other millet varieties, such as foxtail, barnyard, and finger millet, as well as a hybrid of barnyard and pearl millet. Li et al. [33] studied the SP of PMS (2–35%) at 50–90 °C and found that after ultra-high pressure, the treated starch showed lower SP than native starch.

The following solubility of PMS at different temperatures (70–90 °C) was observed for native starch (2.62–34.88%), acid-modified starch (18.97–86.17%), and hydrothermally modified starch (1.71–12.45%) [28]. The solubility of acid-modified starch was higher than that of native starch, which is due to the fact that increasing temperature causes structural weakening and depolarization of starch granules in the former [28]. Li et al. [33] observed the solubility of PMS in a temperature range of 50–90 °C and found that the ultra-high-pressure-treated starch exhibited lower solubility than the native starch at a higher temperature. At 95 °C, Xiao et al. [41] investigated the solubility of native PMS (5.32%) and found a higher solubility of PMS with proanthocyanidins (8.64–16.35%). Wu et al. [29] found that the solubility of PMS was higher than other millets such as foxtail, barnyard, hybrid barnyard, and pearl millets, but lower than finger millet. However, all millet starches exhibited lower SP and solubility patterns in the temperature range of 60–90 °C than other commonly used starch sources (e.g., wheat and potato), suggesting stronger swelling resistance and binding strength within the starch granules [21]. It is thought that the interaction between starch and water molecules upon heating is the cause of the increased solubility and swelling power, and that the starch exposes additional groups that become associated with water molecules [41].

### 4.2. Pasting Properties

In the majority of cases, rheological evaluation of starch was carried out using both the Rapid Visco Analyzer (RVA) and the Brabender Visco-Amylograph (BVA), and the findings are presented in Table 3. This technique involves heating starch with a substantial quantity of water under continuous shear. The viscosity changes at a given temperature cycle are recorded. Pasting is affected by several parameters, including starch structure, water content, temperature program, and shear rate, which are closely monitored. The amount of starch used in the studies we examined ranged from 6 to 10% [6]. Three sections can be identified in a typical pasting curve, each representing a specific phase of starch granule transformation during the pasting process [9]. The first phase involves the gradual absorption of water by the starch granules, causing them to expand; the second phase involves the leaching of the amylose component; and the final phase involves the loss of structural integrity of the expanded starch granules, causing them to disintegrate into fragments [42]. The pasting properties and attributes of starch paste are subject to the influence of several factors, including the concentration of starch, its composition in terms of amylose content and amylose-to-amylopectin ratio, and cooking and cooling temperatures, as well as the presence of solutes such as pH, lipids, and sugars. For instance, waxy starch has a greater tendency to absorb water and expand quickly, enabling it to attain its maximum pasting temperature in a shorter duration as compared to starches with a higher amylose content [43]. Yang et al. [14] reported that the peak viscosity (PV), trough viscosity (TV), and breakdown viscosity (BD) of waxy proso millet starch were greater, while the setback viscosity (SB) and pasting temperature (PT) were lower compared to nonwaxy millet starch. The study conducted on proso millet starch revealed that amylose content had a strong negative correlation with PV, TV, and BD, but a substantial positive correlation with SB and PT. A lower SB indicates better stability, and a lower BD indicates high shear resistance. Waxy proso millet starch demonstrates superior stability, making it a desirable choice for frozen food and thickening applications. On the other hand, nonwaxy proso millet starch exhibits higher temperature stability and improved shear resistance, indicating its potential suitability for medicinal resources [14].

### 4.3. Thermal Properties

The process of gelatinization occurs when starch granules are subjected to a specific temperature range and a sufficient quantity of water, leading to an order–disorder phase transition. Gelatinization is characterized by radial swelling of the granules, water absorption by the amorphous region, leaching of starch molecules, and collapse of the crystalline region with breakup of the double helices [40]. Differential scanning calorimetry (DSC) is most commonly used for the analysis of millet starch, where the initial (*T*_o_), peak (*T*_p_), and conclusion (*T*_c_) gelatinization temperatures, as well as the enthalpy change (Δ*H*), are regularly recorded [6]. Table 4 presents the thermal properties of PMS. The characteristics of gelatinization in starch vary not only between different species of millet, but also among various genotypes within the same species [16]. Various factors, including the granule size and the ratio of amylose to amylopectin, have an impact on the gelatinization properties of diverse types of millet starch. Moreover, these differences are also observed between different varieties of the same plant species. The gelatinization temperature of waxy and low-amylose starches takes a longer time to reach compared to nonwaxy, high-amylose starches [45]. Gelatinization temperatures are also important in the selection of specific starch properties for various food applications [21]. Thermal properties of PMS observed by Yang et al. (2019) [14] in both nonwaxy and waxy starch are as follows: *T*_o_ (64.6–71.1 °C); *T*_p_ (70.5–77.9 °C); *T*_c_ (77.4–82.3 °C); and Δ*H* (9.6–10.8 J/g). A higher gelatinization temperature indicates a perfect crystal structure of starch, while a higher enthalpy indicates that the gelatinization of starch requires more energy [46].

### 4.4. Retrogradation

After gelatinization, when starch is cooled, the amylose and amylopectin molecules bind to each other, leading to recrystallization and forming a more structured organization than previously. Retrogradation is a process, which demonstrates the ability of starch to thicken and create rigid gels. Amylose content, water content, amylopectin’s molecular structure, storage parameters (time, temperature), and the presence of other elements (proteins, fiber, lipids) are all factors that influence retrogradation [40]. Annor et al. [25] reported that retrograded PMS had higher onset gelatinization temperature (*T*_o_) and gelatinization enthalpy (Δ*H*) values than foxtail and pearl millets, but lower than finger millet. Retrograded millet starch, including retrograded pearl millet starch, typically exhibits reduced enthalpies and melting temperatures. This could be due to the fact that the stored gel exhibits unstable recrystallization of the branched chains of amylopectin compared to native gels [16]. Yang et al. [14] stated that retrogradation of waxy PMS increases in the first 2 h and then stabilizes, whereas in nonwaxy PMS it increases in the first 40 h before stabilizing. Retrogradation reflects the stability of a starch, with higher retrogradation indicating deterioration of stability [49]. Li et al. [44] reported both the highest (28.3%) and lowest (0.1%) retrogradation of PMS. These were different cultivars of proso millet with different origins, calling into question a consistent, measurable correlation between locations. Chao et al. [37] conducted a comparative study of waxy PMS and nonwaxy PMS. Their study showed higher retrogradation rates in the first 4 h; after 8 h, the retrogradations slowed down and stabilized after 32 h for both varieties. However, the percentage of retrogradation of waxy PMS was lower than that of nonwaxy PMS. As a crucial factor limiting the use of starch, higher retrogradation may lead to undesirable changes in the biomechanical properties of starch-based foods and affect their nutritional and sensory aspects. For this reason, and because of its better transparency, waxy millet starch with lower retrogradation could be suitable as a raw material source for beverage production [37].

### 4.5. Freeze–Thaw Stability

Starchy foods are exposed to multiple cycles of freezing and thawing during different stages of their preparation and storage. The freeze–thaw stability of starch is evaluated by measuring the quantity of water that separates from starch gels as a result of freeze–thaw cycles (syneresis) [6]. Freeze–thaw stability of starch depends on the amount of amylose and amylopectin, water content, thermal history, and molecular structure. The better freeze–thaw stability of native starch depends on a higher proportion of shorter unit chains of amylopectin and a lower quantity of amylose chains [50]. The stability of the starch gel from proso millet was lower than that of the maize starch gel, but a partially dried starch gel from proso millet could rapidly reabsorb water and restore its original structure [27]. However, Wu et al. [29] reported that the freeze–thaw stability of proso millet starch is much higher than that of other millet species such as foxtail, barnyard, hybrid barnyard, pearl, and finger millets. Therefore, the starch of proso millet is more appropriate for use in the frozen food industry than that of other types of millet.

### 4.6. Digestibility

Starch digestibility is an important nutritional indicator that influences consumer perception of the acceptability or unacceptability of a product [16]. The rate and extent of digestibility, as indicated by time-dependent blood glucose levels in the intestinal tract, determine the starch digestibility factor of a food and are an important metabolic measurement in health care [51]. Rapidly digestible starch (RDS), slowly digestible starch (SDS), and resistant starch (RS) are the three types of digestible starch [52]. RDS is rapidly broken-down during digestion and absorbed by the small intestine. Due to this rapid response, blood glucose levels spike, which in turn increases the likelihood of obesity, type 2 diabetes, and cardiovascular disease [53]. Conversely, SDS takes a long time to be digested. While SDS is also digested in the small intestine, it provides a slower, sustained release of glucose into the bloodstream, resulting in healthier glycemic responses. The low glycemic index of SDS makes it useful in mitigating the risk of obesity and type 2 diabetes, as well as regulating undesirable cycles of excitability and fatigue. SDS is also helpful in many other diseases, such as elevated cholesterol, poor mineral absorption, gallstones, cancer, and reducing fat formation. Conversion of RDS to SDS can have a significant impact on improving the health of millions of people, as it enables a sustained release of glucose into the bloodstream, as opposed to a spiked release. Therefore, it is imperative to include PMS in research trials exploring this area. RS resists breakdown in the small intestine and instead undergoes fermentation in the large intestine, serving as a prebiotic that nourishes beneficial bacteria in the gut [54]. It does not increase blood sugar. Chang et al. [55] reported that nonwaxy proso millet starch has lower RDS content and higher SDS and RS contents than waxy proso millet starch, normal corn starch, and potato starch, indicating a potential functional food formula.

## 5. Starch Modifications

The structure and functions of native starch depend on the plant from which the starch is obtained. There are some limitations of native starch that restrict its direct application in the food and nonfood industry, especially the low thermal stability and shear strength and the high degree of retrogradation [56]. Notwithstanding these poor gelling properties of their pastes, native starches may also be prone to syneresis, which further reduces water content by increasing coagulation. To address this deficiency of native starch, physical, chemical, and enzymatic processes are commonly used in the food, paper, and textile industries to stimulate and enhance certain functional properties of native starch, including PMS [21]. Derivatization (e.g., etherification, esterification, and crosslinking), disintegration (by acid hydrolysis and oxidation of starch), enzymatic treatments (e.g., fermentation), and physical modifications (e.g., heat–moisture treatment (HMT) and ultra-high-pressure treatment (UHP)), or combinations of these, are the most commonly used methods for starch modification [16].

### 5.1. Acid Treatment

Acid modification is a chemical process used to modify starch that involves hydrolysis of the α-glucan chains through the use of mineral acids. This process results in the breaking of glycosidic bonds, leading to the alteration of the structure and properties of native starch [57]. Acid hydrolysis is a widely utilized process for the modification of starch. It involves the exposure of starch to mineral acids, such as HCl, H_2_SO_4_, H_3_PO_4_, and HNO_3_, resulting in an increase in the number of short starch chains, as found in amylose. Despite being commonly employed, the primary limitation of this technology is its heavy reliance on chemical agents, which can result in adverse environmental impacts [16]. In addition to altering the structure of native starch, acid modification also changes its physicochemical properties, making it suitable for use in various industries such as food and textiles. This modified starch is utilized in the production of starch gum candy, paper, and cationic and amphoteric starches, among other applications [21]. Compared with native PMS, acid-modified starch reduces water binding capacity. It is suggested that this reduction is due to the fact that acid modification reduces the size of the amorphous region, thereby reducing accessibility to the binding sites [28].

### 5.2. Hydrothermal Treatment

Heat–moisture treatment (HMT) is a hydrothermal processing method carried out under high temperatures ranging from 90–120 °C, and low moisture levels of 35% or below. The process of HMT involves subjecting starch to a controlled temperature above its glass transition temperature for a specified duration, typically between 1 to 24 h, to achieve a low moisture content. The period of the procedure is dependent on the desired outcome and specific treatment process [58]. Annealing (ANN) refers to the hydrothermal process of heating starch with a water content of 40–65% at temperatures lower than the onset of gelatinization. This treatment facilitates the interaction and reassociation of amylose and amylopectin chains inside the starch granules, leading to the repair of structural defects in the crystalline portion [6]. HMT decreases the swelling power, the solubility, the PV, the BD and SB values, and the gelatinization enthalpy of PMS, while the pasting and gelatinization temperatures increase [59,60]. A reduction in the swelling power is desirable for some applications such as noodle production, while a reduction in the breakdown and setback values improves the hot and cold paste stability, respectively, of PMS. Kumar et al.’s [59] findings indicate that the in vitro digestibility of PMS demonstrated an increase in the rapidly digestible starch (RDS) and slowly digestible starch (SDS) fractions, with a decrease in the resistant starch (RS) fraction after undergoing HMT. The effect of HMT on enzymatic digestibility of starch is influenced by various factors such as (i) type of starch source, (ii) moisture content, (iii) treatment temperature and duration, and (iv) interactions between different starch fractions, including amylopectin–amylopectin, amylose–amylose, and amylose–amylopectin interactions [61,62].

### 5.3. Dry Heat Treatment (DHT)

DHT is a form of physical modification that induces alterations in the physicochemical properties of starch, while preserving its granular structure. Dry heat represents a simple, nontoxic, and healthful substitute for chemical-based techniques. The new properties that occur with thermally treated starch are similar to the results of the chemical crosslinking process. Dry heat improves the pasting and functional properties of starch as effectively as the chemical alternative [22]. In the case of proso millet starch, DHT (8% moisture content, 130 °C, 2 and 4 h) resulted in a decrease in peak (PV) and breakdown (BD) viscosities and pasting temperature (PT) and an increase in final (FV) and setback (SB) viscosities compared to the native starch, the extent of which increased with increasing treatment time. The decrease in BD demonstrated that DHT-modified starch became more resilient to thermal and mechanical shear, thus exhibiting heightened hot paste stability comparable to that of chemically crosslinked starch. The DHT starch could be used in the products which require higher final viscosity and hot paste stability. DHT of PMS also led to an increase in the onset (*T*_o_) and peak (*T*_p_) gelatinization temperatures and a decrease in gelatinization enthalpy (Δ*H*). A decrease in Δ*H* could be attributed to a decrease in crystallinity in the starch granule after DHT, which was determined by X-ray diffraction [22].

### 5.4. Ultra-High-Pressure (UHP) Treatment

UHP is a nonthermal modification approach that can be employed to induce gelatinization or physical modification of diverse starch types. The degree of gelatinization attained through UHP, as an alternative to conventional thermal processing, is influenced by several factors, including starch type, pressure levels, water content, temperature ranges, and duration of treatment. UHP completely gelatinizes starch when it is under sufficient pressure at specified ambient temperatures [33]. The use of UHP to change the composition of millet starch or its molecular structure has hardly been explored so far. With the exception of proso millet starch treated at a maximum pressure of 600 MPa for 15 min, which caused a decrease in all viscosities of the paste during pasting, the application of UHP at different pressures between 150 and 450 MPa increased the trough and final viscosities and the pasting temperature, but decreased the peak and breakdown viscosities compared to native starch [33]. The exerted pressure potentially enhanced the infiltration of water molecules into the starch granules, disrupted hydrogen bonds, and instigated alterations in the starch configuration. The pasting properties of starch treated at 600 MPa were dissimilar to those of starch treated at 150–450 MPa, implying complete gelatinization of the starch at higher pressure [63,64].

### 5.5. Fermentation

Through the process of fermentation, it is feasible to alter the chemical and physical characteristics of starch. This methodology serves to augment the structural attributes of starch. Bian et al. [65] conducted a study on the impact of lactic acid bacteria fermentation on the physical properties and structure of glutinous (waxy) PMS. During fermentation, microbial enzymes and acids hydrolyze the starch molecules predominantly located in the amorphous region into smaller molecules. The fermented PMS had higher amylose content and crystallinity, but lower molecular weight, swelling power, and solubility than the nonfermented PMS. All RVA pasting parameters of PMS decreased after fermentation, except for the setback viscosity (SB), which increased. The gelatinization enthalpy (Δ*H*) of the fermented PMS increased compared to that of the nonfermented PMS, reflecting the increase in crystallinity after fermentation.

### 5.6. Dual Modifications

Dual modification of starches is gaining popularity in current scientific research. This is because single modification methods may not always fulfill the necessary functional requirements for food and industrial applications. Dual modification of starch offers the ability to customize starch properties for specific applications and to enhance the functionality of single modified starch, thereby increasing the range of applications for starch. Sun et al. [66] studied the effects of dual physical modification with UHP and cold plasma (CP) on the properties and digestibility of PMS. CP is an environmentally friendly and nonthermal method that utilizes gas ionization to produce various free radicals which react with starch, leading to either crosslinking or depolymerization of the starch without the use of chemicals [67]. Application of dual modification with UHP at 600 MPa and CP resulted in gelatinization and depolymerization of PMS and an elevated content of resistant starch (RS). In addition, the dual modified starch exhibited lower breakdown viscosity, indicating higher stability of the paste to heat and mechanical shear [66].

## 6. Potential Applications and Future Perspectives

Starch is a versatile biomaterial with a wide range of applications across the globe in industries such as food, pharmaceuticals, textiles, and various other industrial sectors. The physicochemical and functional properties of starch determine its role in several industries. Both native and modified forms of millet starch are utilized in several industries, especially in the food industry. However, native millet starch has limited functionality and, as a result, has fewer industrial applications. Native and modified starches are commonly employed in the food industry as thickeners, binders, fat substitutes, gelling agents, and stabilizers for emulsions and foams [16]. The improved functional properties of modified PMS and its potential applications can be summarized as follows: (1) reduction of swelling power and solubility by HMT, UHP, and fermentation to increase tensile strength, firmness, and minimize cooking losses (solubility) of pasta, noodles, and edible films, (2) reduction of breakdown (BD) viscosity by HMT, DHT, UHP, and dual UHP/CP to increase shear and cooking stability of thickeners, sauces, confectionery, and canned products, (3) reduction of setback (SB) viscosity by HMT to reduce staling and syneresis of bakery and frozen products, respectively, (4) increase in final viscosity (FV) by DHT and UHP to form a viscous paste or gel after cooking and cooling, which is essential for thermal processing such as canning, and (5) increase in the content of SDS and/or RS by HMT and dual UHP/CP for use as a functional food ingredient. In addition, PMS proved to be very suitable for nanoparticle formation after modification with the enzyme pullulanase [68] and ball-milling [69]. Nano-reduction of PMS improved the antioxidant activity of PMS and made it an effective functional food ingredient that can be used in commercial applications. The unconventional and underutilized starches have become the focus of research in recent years due to their technical advantages over conventional starches and can be used in various areas of the food and nonfood industry [70]. The potential applications of PMF and PMS are summarized in Table 5.

## 7. Conclusions

Millet is a crop that is typically grown in semiarid regions where traditional cereals struggle to grow. It possesses a wealth of health-promoting properties that are unfortunately neglected, resulting in millet not being widely accepted as a food in its own right by the commercial food industry. Starch is a key component of millet, and yet this too is underutilized as a source in preference to other conventional sources of starch. The functional properties of millet starch are similar to those of other starches, as it can act as a structure provider, texture modifier, binder, and viscosity regulator.

PMS has been underutilized and under-researched in comparison to major cereal and tuber starches that have been extensively studied. Research carried out on the modifications of starch depends on the wide range of targeted consumer products by for-profit companies and is motivated by desired functionalities rather than commercial distribution of the starch. Research on the modification of PMS using such methods as chemical, physical, and enzymatic treatments (or a combination of these methods) still have areas that remain to be investigated; due to this, its commercial application is disproportionately limited given its equivalent biological performance. Physical modification of all starches has gained popularity in recent years because it is nontoxic, hygienic, and environmentally friendly. In the search for better methods, novel physical modifications such as pulsed electric field processing, ultrasonication, cold plasma treatment, and irradiation can be used. This provides the opportunity to explore the physicochemical properties of PMS (e.g., freeze–thaw stability, rheology, gel properties, and retrogradation) and categorize them using laboratory research methods and the full range of our advanced scientific instruments to reveal the unique properties of this starch. While it is acknowledged that PMS can be used as a stabilizer, thickener, and fat substitute in various food products, there is still room for further exploration of its potential applications in the food industry. In particular, there is potential for increased use of PMS in the formulation of functional foods as well as in products such as bakery items and pasta. Beyond the food sector, PMS can also be used in other industries, and a comparative study with the predominantly used conventional starches will certainly open up new areas of functionality.

Therefore, PMS is an excellent alternative for the many industrial applications currently covered by the more widely used starches (e.g., corn, wheat, cassava, and potato). Due to its initial poor functionality and lack of known modifications, PMS is currently rarely used in its original form. However, various agencies and research studies have found that starch modification can produce the desired properties and characteristics in the targeted foods. All starches are rarely used in their native form due to their low functionality, and most starches must be modified prior to commercial use (as all current modification methods are known to be safe and effective for their respective purposes). This is similar to the circumstances surrounding PMS and cannot explain the clear bias against PMS. At this point, it can also be noted that all future research will bring newly discovered modifications that can make new contributions to structural changes in starch. This, in turn, could change a negative starch property for the better.

These treatments, past, present, and future, have the common denominator of making structural changes to starch and bringing benefits to our modern lifestyle. The connection between the native starch properties and the modified starch structure can be better understood with the use of several techniques such as spectroscopy, microscopy, XRD, and calorimetry. Currently, access to these scientific tools for PMS is limited. The information that will result from greater access to scientific methods will contribute to the development of PMS-based foods that are beneficial to both lifestyle and health. This review provided a comprehensive overview of the properties of PMS; by highlighting the value-added uses of PMS, its equivalent properties, and its potential future advances, PMS can be developed as a highly utilized starch in related industries.

## Figures and Tables

**Table 1:** Starch yield and chemical composition of proso millet starch.

Source	Starch Yield (%)	Protein (%)	Amylose (%)	Lipid (%)	Reference
Proso millet	93.7	-	33.9	-	[25]
	92.19–94.60	0.27–0.67	14.92–17.37	0.13–0.25	[26]
	-	0.69–4.31	27.2–29.1	0.59–0.6	[23]
	61.8–68.2	1.1–2.1	29.2–32.6	-	[27]
	87.27–94.60	1.07–1.30	2.80–32.80	0.01	[14]
	-	0.48	1.61	0.01	[22]
	54.1	1.21	28.51	0.27	[28]
	-	0.45	0.38	-	[29]

**Table 2:** Proso millet starch’s native and modified morphological properties.

Starch Source	Type	Size (µm)	Shape	Reference
Proso millet	NS	3–10	Oval, polygonal, irregular, and spherical	[33]
	UHP	-	Structural disruption, gel-like structure formed	[33]
	NS	2.5–17	Few spherical and mostly polygonal	[25]
	NS	0.3–12	Few small spherical granules and mainly uniform large or small polygonal	[26]
	NS	3–10	Few small spheres and large polygonal shape	[29]
	NS	4.3–8.9	Mostly polygonal with some elliptical granules having rounded edges and surface pores	[34]
	NS	5–12	Round and smooth	[22]
	DHT	-	Smooth and plump surface with large lumps	[22]
	NS	1.8–13.5	Bimodal distribution, small spherical and large polygonal	[27]
	NS	1.3–8	Bimodal distribution, large polygonal, small and large spherical	[35]
	NS	4.49–4.70	Regular, polygonal, and round shape, along with the characteristic Maltese cross structure	[14]
	NS	1.54–11.7	Mainly polygonal and round shape, larger and smaller granules make honey-comb structure	[9]

Key: NS, native starch; DHT, dry-heat-treated; UHP, ultra-high-pressure-treated.

**Table 3:** Pasting properties of proso millet starch.

Starch (g/mL)	Method	Unit	PV	BD	SB	PT (°C)	Reference
-	BVA	BU				72.5–74.5	[27]
-	BVA	BU	520	50	330	75.8	[35]
3.5/25	DHR	Pa.s	4.60	2.60	1.69	79.23	[28]
3/25	RVA	cP	2807	1746	1634	57.40	[33]
3/25	RVA	cP	2372	1792	582	-	[29]
3/25	RVA	cP	2822	1854	501	76	[22]
2.5/25	RVA	cP	2284.5	913	372.5	79.18	[41]
2/25	RVA	cP	2134–3515	488–967	197–1102	63.60–63.80	[37]
3/25	RVA	cP	2110–3286	1114–2189	279–1478	77.8–80.9	[14]
2/26	RVA	cP	2215–3585	511–1437	752–1435	78.8–82.8	[44]
-	BVA	BU	219–457	79.5–240	115.05–201.5	-	[26]

Key: RAV = Rapid Visco-Analyzer, DHR = Discovery Hybrid Rheometer, BVA = Brabender Visco-Amylograph. The viscosity units for RVA, BVA, and DHR are cP, BU, and Pa.s, respectively; PV = peak viscosity; BD = breakdown viscosity; SB = setback viscosity; PT = pasting temperature (°C).

**Table 4:** Thermal properties of proso millet starch determined by differential scanning calorimetry (DSC).

Starch Water Ratio (*w*/*w*)	Heating Rate (°C/min)	*T*_o_ (°C)	*T*_p_ (°C)	*T*_c_ (°C)	Δ*H* (J/g)	References
1:3	5	68.4	72.2		13.1	[25]
1:3	10	72.7–73.6	75.8–77.6	84.4–89.5	13.2–14.8	[23]
1:2	5		65.8–80.2		6.4–11.4	[47]
1:2.7	10	67.8–69.0	69–73.9	75.5–81.8	13.2–14.8	[27]
1:2	5	62–69	67–74	77–78	9.6–12.6	[48]
1:2	10	68.65	71.37	80.04	15.03	[22]
1:2	10	71.95	77.36	87.42	14.98	[41]
1:2	10	68.56	74.53	82.43	5.16	[29]
1:3	10	73.1–76.4	78.0–81.5	79.3–86.0	0.81–4.48	[34]
1:3	10	67.4–75.5	71.5–79.0	76.5–84.0	11.9–17.6	[44]
1:3	10	66.81–70.01	72.79–76.55	78.30–82.44	10.40–14.46	[26]
1:2	10	64.6–71.1	70.5–77.9	77.4–82.3	9.6–10.8	[14]
1:3	10	67.9–72.7	74.6–76.1	80.4–81.2	10.37–12.65	[37]
1:2	10	72.93	78.61	94.55	3.83	[28]
1:4	10	64.16	68.45	79.09	10.58	[33]

**Table 5:** Applications of proso millet flour and starch.

Proso Millet Type	Characteristics	Applications	References
Native PMS and PMS-k-carrageenan blend	The PMS film showed higher antioxidant activity than the PMS-k-carrageenan blend film but lower water permeability and solubility.	Edible film	[71]
Native PMS with curcumin (0–3%)	The antioxidant activity and the water and light barrier properties of the PMS film increased with increasing curcumin concentration.	Packaging film	[72]
Native PMS	The total film produced using native PMS proved to be a suitable packaging material for food products.	Packaging film	[73]
Native waxy and non-waxy PMS	RS content of waxy and non-waxy PMS was increased by HMT.	Functional foods	[74]
Native proso millet flour	Incorporation of hydrocolloids and proso millet flour into corn-based noodles resulted in enhanced texture and nutrition attributes of the noodles.	Gluten-free noodles	[75]
Native proso millet flour	Incorporation of proso millet flour to wheat flour muffins improved nutritional quality and antioxidant activity.	Bakery products	[76]
HMT proso millet flour	Incorporation of HMT proso millet flour to gluten-free millet cake improved the quality of the cake.	Gluten-free bakery products	[77]
Native PMS	The use of PMS as a thickener in textile printing improved the quality of the print.	Textile printing	[78]
Native proso millet flour	The quality of bread made from a wheat/proso millet mixed flour (50:50) was improved to be acceptable by addition of emulsifier and enzymes.	Bakery products	[79]

## Data Availability

Not applicable.
